# Ultrasonographic measurement of the optic nerve sheath diameter and its association with eyeball transverse diameter in 585 healthy volunteers

**DOI:** 10.1038/s41598-017-16173-z

**Published:** 2017-11-21

**Authors:** Dong Hwan Kim, Jin-Sun Jun, Ryul Kim

**Affiliations:** 1 Department of Radiology, Aerospace Medical Center, Republic of Korea Air Force, 635 Danjae-ro, Namil-myeon, Cheongwon-gun, Chungcheongbuk-do 363-849 Republic of Korea; 2 Department of Neurology, Seoul National University Hospital, College of Medicine, Seoul, Korea; 3 Department of Neurology, Aerospace Medical Center, Republic of Korea Air Force, 635 Danjae-ro, Namil-myeon, Cheongwon-gun, Chungcheongbuk-do 363-849 Republic of Korea

## Abstract

The optic nerve sheath diameter (ONSD) is considered as an indirect marker for intracranial pressure (ICP). However, the optimal cut-off value for an abnormal ONSD indicating elevated ICP and its associated factors have been unclear. Thus, we investigated normative values for the ONSD using ultrasonography and investigate the potential factors affecting it. We prospectively recruited healthy volunteers between September 2016 and March 2017. A total of 585 individuals were included, in which the mean ONSD was 4.11 mm [95% confidence interval (CI), 4.09–4.14 mm]. Although ONSD was correlated with sex (*p* = 0.015), height (*p* = 0.003), and eyeball transverse diameter (ETD) (*p* < 0.001) in simple linear regression analyses, multiple linear regression analysis revealed that only ETD was independently associated with ONSD (*p* < 0.001). Accordingly, we further established a normative value for the ONSD/ETD ratio and its associated factors. The mean ONSD/ETD ratio was 0.18 (95% CI, 0.18–0.18), but the ONSD/ETD ratio was not correlated with sex, height, weight, body mass index, and head circumference. Our findings suggest that the ONSD had a strong correlation with ETD, and ONSD/ETD ratio might provide more reliable data than ONSD itself as a marker of ICP.

## Introduction

Elevated intracranial pressure (ICP) is a potentially devastating condition resulting from various neurological and non-neurological disorders^1^. Rapid detection of elevated ICP and subsequent management are important because it is associated with poor prognosis^2,3^. The golden standard for estimating ICP includes invasive methods such as intraventricular catheterization and intraparenchymal probes^4^. However, such procedures are not routinely performed because of the absence of neurosurgeons or intensive care units and the risk of complications including hemorrhage and infection. In addition, they are contraindicated in patients with thrombocythemia or coagulopathy^5,6^. Accordingly, the importance of non-invasive methods for ICP measurement has increased.

The optic nerve sheath diameter (ONSD) is considered as an indirect marker for ICP estimation^7^. Measurement of ONSD by ultrasonography is a rapid and easily accessible bedside test. Furthermore, growing evidence has shown that this procedure has high reproducibility and low intra- and inter-observer variability^8,9^. Given that computed tomography (CT) or magnetic resonance imaging is time consuming and requires patient transfer, ultrasonographic measurement of ONSD may be a good choice for the detection of elevated ICP in clinical settings and research. However, despite its usefulness and popularity, the optimal cut-off value for an abnormal ONSD indicating elevated ICP has been unclear, because most studies on ONSD measurement included only a small number of healthy individuals. Furthermore, although previous studies investigated demographic and physiological factors associated with ONSD, the results have been inconsistent or inconclusive. A clear understanding of the normal range for ONSD and its associated factors is crucial to interpret the measurement as a marker of ICP.

Therefore, the main aims of the current study were to establish normative values for ONSD using ultrasonography in a large number of healthy Korean adults and investigate potential factors affecting this parameter.

## Materials and Methods

### Study Population

We prospectively recruited healthy volunteers who visited the Republic of Korea Air Force Education And Training Command for physical examination required to become a soldier between September 2016 and March 2017. All volunteers were limited to young healthy adults aged 18–30 years, given the nature of the military organization. We excluded individuals with a history of neurological disorders. Written informed consent was obtained from all participants before enrollment. The study protocol was approved by the Institutional Review Board of the Armed Forces Medical Command (Seongnam, Korea) and followed to the principles of the Declaration of Helsinki.

The following data were recorded for each subject: age, sex, height, weight, and head circumference. The head circumference was measured by a single investigator (R.K., a board-certified neurologist) using a nonstretchable tape around the widest possible occipitofrontal circumference. The body mass index (BMI) was calculated as the weight in kilograms divided by the height in meters squared.

### Ultrasound Measurement

Ultrasonic examination was performed by an experienced investigator (D.H.K., a board-certified radiologist) using a GE Logiq P6 scanner (General Electrics Medical Systems, Milwaukee, WI, USA) with a 11–3 MHz linear transducer. The subjects were examined in a supine position with the head elevated at 20–30°. They were instructed to keep their eyes shut in a mid-position of the bulb and suppress any eye movement. A thick layer of conductive ultrasound gel was applied over the closed upper eyelid. The probe was placed gently on the gel in the temporal area of the eyelid to prevent pressure from being exerted on the eye.

**Figure 1:**
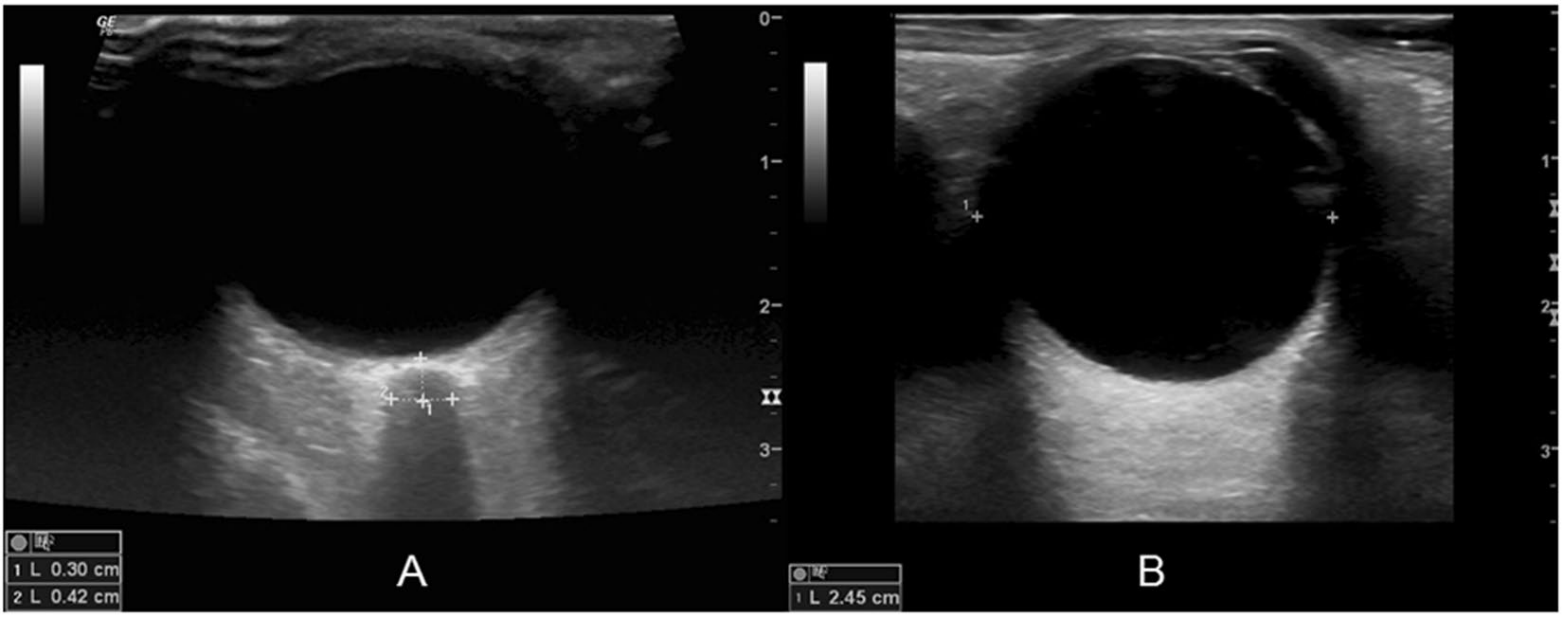
A sample ultrasonographic image for estimation of the ONSD (**A**) and ETD (**B**). (**A**) ONSD (measurement “2”) is measured 3 mm behind the globe using an electronic caliper along the axis (measurement “1”). *Abbreviation*s: ONSD, optic nerve sheath diameter; ETD, eyeball transverse diameter.

ONSD and the eyeball transverse diameter (ETD) were measured for each eye in the horizontal plane (Fig. 1). ONSD was defined as the distance between the external borders of the hyperechoic area 3 mm posterior to the point where the optic nerve entered the globe, using an electronic caliper along the axis perpendicular to the retina. ETD (retina to retina) was defined as the maximal transverse diameter of the eyeball obtained by scanning from the superior to the inferior side. To minimize intraobserver variability, each measurement was performed three times and the mean value was derived. Before the enrollment of participants, a training session with 15 healthy volunteers who were not included in this study was held to familiarize the examiner with the ultrasonic measurement of ONSD and ETD.

### Statistical Analysis

Continuous variables are presented as medians and interquartile ranges (IQRs) or means ± standard deviations (SDs), while categorical variables are reported as frequencies and percentages. Continuous variables were compared using paired *t*-tests. We used a series of simple linear regression models to identify demographic and physiological factors associated with ONSD. Potential factors of interest were selected a priori on the basis of previous literature; these included sex, height, weight, BMI, head circumference, and ETD. The factors that survived simple linear regression analyses were then evaluated using multiple linear regression models. A two-tailed *p*-value of <0.05 was considered statistically significant. All statistical analyses were performed with IBM SPSS version 18 (IBM Software Inc.).

## Results

In total, 585 healthy volunteers were included in this study. The mean age was 21.4 ± 1.9 years, and 508 (90.4%) subjects were men. The mean height, weight, BMI, and head circumference of the individuals were 171.1 ± 6.8 cm, 68.5 ± 11.6 kg, 22.8 ± 3.1 kg/m^2^, and 57.6 ± 1.7 cm, respectively.

ONSD and ETD values are detailed in Table 1. ONSD ranged from 3.30 mm to 5.20 mm, and 95% subjects exhibited a mean ONSD in the range of 4.09 mm to 4.14 mm. The median ONSD was 4.10 mm (IQR, 3.85–4.35 mm). There was no significant difference in ONSD between the right and left eyes (*p* = 0.510). The median and mean ETD were 22.85 mm (IQR, 22.25–23.45 mm) and 22.91 ± 0.93 mm, respectively.

**Table 1:** Basic descriptive statistics for ONSD, ETD, and the ONSD/ETD ratio measured using ultrasonography for healthy Korean adults.

	Mean ± SD (95% CI)	Median (IQR)	Minimum	Maximum
ONSD				
Right (mm)	4.12 ± 0.37 (4.09–4.15)	4.10 (3.80–4.40)	3.10	5.20
Left (mm)	4.11 ± 0.37 (4.08–4.14)	4.10 (3.80–4.30)	3.20	5.40
Overall (mm)	4.11 ± 0.35 (4.09–4.14)	4.10 (3.85–4.35)	3.30	5.20
ETD				
Right (mm)	22.95 ± 0.95 (22.87–23.02)	22.90 (22.30–23.50)	20.90	25.60
Left (mm)	22.87 ± 0.95 (22.79–22.94)	22.80 (22.20–23.40)	20.70	25.90
Overall (mm)	22.91 ± 0.93 (22.83–22.98)	22.85 (22.25–23.45)	20.90	25.70
ONSD/ETD ratio				
Right	0.18 ± 0.02 (0.18)	0.18 (0.17–0.19)	0.13	0.23
Left	0.18 ± 0.02 (0.18)	0.18 (0.17–0.19)	0.14	0.24
Overall	0.18 ± 0.02 (0.18)	0.18 (0.17–0.19)	0.14	0.23

The overall ONSD value is the mean of the values for the left and right eyes.

ONSD, optic nerve sheath diameter; ETD, eyeball transverse diameter; SD, standard deviation; CI, confidence interval; IQR, interquartile range.

The results of regression analyses are summarized in Table 2. Simple linear regression analyses showed that ONSD was correlated with sex (*p* = 0.015), height (*p* = 0.003), and ETD (*p* < 0.001). After adjustment for potential confounders between these factors, only ETD was found to be independently associated with ONSD (*p* < 0.001). Accordingly, we further analyzed normative values for the ONSD/ETD ratio and its associated factors including sex, height, weight, BMI, and head circumference. The ONSD/ETD ratio ranged from 0.14 to 0.23, and 95% subjects exhibited a mean ONSD/ETD ratio of 0.18. There was no significant difference in the ONSD/ETD ratio between the right and left eyes (*p* = 0.329). Simple linear regression analyses showed that the ONSD/ETD ratio was not correlated with sex (*p* = 0.140), height (*p* = 0.505), weight (*p* = 0.826), BMI (*p* = 0.516), and head circumference (*p* = 0.387).

**Table 2:** Finding of linear regression analyses for potential factors associated with ONSD.

Variables	Simple linear regression analysis			Multiple linear regression analysis		
	B-Coefficient	SE	P	B-Coefficient	SE	*P*
Sex	−0.121	0.050	0.015	−0.069	0.057	0.224
Height	0.006	0.002	0.003	0.003	0.002	0.310
Weight	0.002	0.001	0.098	—		—
BMI	0.002	0.005	0.683	—		—
HC	0.012	0.008	0.171	—		—
ETD	0.088	0.015	<0.001	0.083	0.016	<0.001

ONSD, optic nerve sheath diameter; BMI, body mass index; HC, head circumference; ETD, eyeball transverse diameter; SE, standard error.

## Discussion

In the present study, we assessed normative values for ONSD and its associated factors using ultrasonography in 585 healthy Korean adults. The mean [95% confidence interval (CI)] values for ONSD, ETD, and the ONSD/ETD ratio were 4.11 ± 0.35 mm (4.09–4.14 mm), 22.91 ± 0.93 mm (22.83–22.98 mm), and 0.18 ± 0.02 (0.18–0.18), respectively, and ONSD was associated with ETD, but not with sex, height, weight, BMI, and head circumference. To our knowledge, the present study included the largest number of healthy volunteers among all studies evaluating ONSD using ultrasonography. Therefore, we believe that our findings regarding the normal range for ONSD and its associated factors are reliable.

The mean ultrasonographic ONSD in the current study was 4.11 mm (range, 3.3–5.2 mm); this value is close to that reported by Lee *et al*.^10^, who reported a mean CT-based ONSD of 4.1 mm (range, 2.9–5.3 mm) in the Korean population. The normal range for ultrasonographic ONSD in healthy volunteers has been reported in many other countries (Table 3)^8,9,11–29^. Despite the diversity in the results of these studies, the ONSD measurements in this study were within the normal range of previously reported values. In addition, the upper normal ONSD limit (5.2 mm) and the upper bound of the 95% CI (4.14 mm) for the mean ONSD value corroborated with the optimal cut-off value for the identification of elevated ICP suggested by previous studies where the lowest bound of ONSD values was ≥5.2 mm in patients with elevated ICP^30–36^. Although some authors insist that ONSD is influenced by ethnicity, we could not find obvious differences between Korea and other countries^13,14,16,35,37^.

**Table 3:** Summary of published studies estimating ONSD using ultrasonography in healthy volunteers.

Author (Reference)	Nation	Number^*^ [mean age (years)]	Mean ± SD (95% CI)	Range	Associated factor	Non-associated factor
**Asian population**						
Maude^11^	Bangladesh	136 (NA)	4.41 (4.25–4.75) mm	4.24–4.83 mm	NA	Sex, age, HC
Shirodkar^12^	India	41 (27.4)	F: 4.63 ± 0.09 (4.59–4.67) mm	NA	NA	NA
			M: 4.80 ± 0.10 (4.76–4.84) mm			
Chen^13^	China	519 (46.1)	5.1 ± 0.5 (5.06–5.14) mm	3.5–6.4 mm	OND	Sex, age, weight, height, ETD
Wang^14^	China	230 (43.2)	3.46 ± 0.28 (3.42–3.49) mm	2.65–4.30 mm	Sex, BMI	Age, HC, waistline, MABP
Karami^15^	Iran	32 (59.5)	3.2 ± 0.3 (3.1–3.3) mm	2.6–4.1 mm	NA	NA
Rehman^16^	Pakistan	26 (34.7)	4.33 ± 0.38 (4.18–4.48) mm	NA	NA	NA
Current study	South Korea	585 (21.4)	4.11 ± 0.35 (4.09–4.14) mm	3.30–5.20 mm	ETD	Sex, weight, height, BMI, HC
**Western population**						
Ballantyne^17^	UK	67 (37)	3.2–3.6 mm	2.4–4.7 mm	NA	NA
Romagnuolo^18^	USA	10 (NA)	Rt.: 4.6 ± 0.71 (4.09–5.11) mmLt.: 4.5 ± 0.56 (4.1–4.9) mm	NA	NA	Position
Blehar^19^	USA	27 (36.6)	4.3 (4.0–4.7) mm	NA	NA	NA
Shah^20^	USA	40	3.71–3.92 mm	NA	NA	NA
Skoloudik^21^	Czech Republic	16 (68.6)	3.41 mm	NA	NA	NA
Bauerle^8^	Germany	40 (37.1)	5.4 ± 0.6 (5.2–5.6) mm	4.3–7.6 mm	NA	Sex, age, BMI
Bauerle^22^	Germany	15 (24.5)	5.43 ± 0.49 (5.18–5.68) mm	4.6–6.4 mm	NA	NA
Strapazzon^23^	Italy	19 (39.5)	5.45 ± 0.29 (5.32–5.58) mm	4.85–5.94 mm	Hypobaric hypoxia	NA
Lochner^24^	Italy	21 (34.2)	Median: 5.2 (IQR: 4.8–5.5) mm	NA	NA	Sex, age
Lochner^9^	Italy	20 (46.3)	5.95 ± 0.68 (5.65–6.25) mm	4.5–7.7 mm	NA	NA
Amini^25^	USA	42 (24)	Rt.: 4.73 ± 0.73 (4.50–4.96) mm Lt.: 4.48 ± 0.62 (4.28–4.68) mm	NA	NA	NA
Lefferts^26^	USA	20 (24)	4.99 ± 0.68 (4.69–5.29) mm	NA	NA	Acute resistance exercise
Goeres^27^	Canada	120 (29.3)	3.68 ± 0.36 (2.85–4.40) mm	NA	Sex	Age, weight, height
Zeiler^28^	Canada	120 (29.3)	3.68 (2.85–4.40) mm	NA	NA	NA
Dinsmore^29^	Canada	11 (33.5)	4.2 ± 0.7 (3.8–4.6) mm	NA	End-tidal PCO_2_	NA

^*^Data are number of healthy volunteers.

ONSD, optic nerve sheath diameter; SD, standard deviation; CI, confidence interval; NA, not applicable; HC, head circumference; OND, optic nerve diameter; ETD, eyeball transverse diameter; BMI, body mass index; MABP, mean arterial blood pressure; IQR, interquartile range.

There was a strong correlation between ONSD and ETD in the present study, and this finding is supported by a previous study evaluating CT-based ONSD^38^. Several studies have assessed the relationship between sex and ONSD, although the results have been inconsistent (Table 3)^8,11,13,14,24,27^. We found an association between sex and ONSD in our simple regression analyses but not in our multiple regression analyses including ETD. This finding strongly suggests that sex is not associated with ONSD, and ETD is a confounding factor for their relationship. Weight was not associated with ONSD in the current study, which is consistent with the results of previous studies (Table 3)^13,27^. On the other hand, Wang *et al*.^14^ reported that ONSD was associated with BMI in a study of 230 healthy individuals; however, no significant association was found between the two variables in our larger-scale study.

To the best of our knowledge, the present study is the first to establish a normal value for the ONSD/ETD ratio using ultrasonography. Our derived ratio is very similar to the CT-based ratio of 0.19 derived by Vaiman and Beckerman *et al*.^38^, who also found that the ONSD/ETD ratio was significantly higher in patients with intracerebral hemorrhage than in healthy individuals and exhibited a reasonably good correlation with invasive ICP values^39,40^. The ultrasonographic ONSD/ETD ratio may be a better marker of elevated ICP compared with ONSD alone for several reasons. First, there was a strong correlation between ONSD and ETD. Second, the standard deviation between the normative and pathological values for ONSD was overlapping. Finally, the ONSD/ETD ratio was independent of demographic factors such as sex, height, weight, and BMI. Further trials are warranted to validate this parameter in patients with elevated ICP.

There have been technical issues associated with ultrasonographic measurement of the ONSD. Although some authors have suggested that B-mode ultrasound scan is less accurate in evaluating ONSD owing to blooming effect^41–45^, we used this mode in the current study because in standardized A-mode scan, it is difficult to choose a distance behind the globe in order to consistently measure the nerve^7^. Moreover, B-mode scan is easier to use compared to the A-mode scan and can be used even for patients with a reduced level of cooperation. For these reasons, B-mode scan is more commonly used in the emergency room and intensive care unit^7,46^. The other technical issue is that measurement of the ONSD using B-mode scan may be more accurate in lateral gaze position (abduction) given the poor lateral resolution^47^. However, these eye movements can be performed only in well-coordinated individuals. Unfortunately, patients with suspected elevated ICP are either unconscious or poorly coordinated in many cases. In addition, Lagreze *et al*.^48^ reported no significant difference between the results of the straight and lateral gaze tests with B-mode ultrasound in healthy subjects. Thus, we measured the ONSD in the straight gaze position.

This study has some limitations. Ultrasonographic measurements were obtained by a single experienced board-certified radiologist; therefore, interobserver variability was not evaluated in this study. However, each measurement was obtained three times to minimize errors and bias. In addition, all volunteers were limited to young healthy adults; therefore, the generalizability of our findings to populations of all ages is limited. However, many previous studies have reported that ONSD is not associated with age, and some authors have suggested that ONSD remains more or less constant during the life of an individual^8,11,13,14,24,27^. Accordingly, this limitation is not likely to be significant.

In conclusion, we found that the mean ONSD and the ONSD/ETD ratio determined using ultrasonography in healthy Korean adults were 4.11 mm (95% CI, 4.09–4.14 mm; IQR, 3.85–4.35 mm) and 0.18 (95% CI, 0.18–0.18; IQR, 0.17–0.19), respectively. ONSD exhibited a strong correlation with ETD, but not with sex, height, weight, BMI, and head circumference. Our findings suggest that the ONSD/ETD ratio measured using ultrasonography may be a more accurate and helpful marker of elevated ICP compared with ONSD alone, which is further investigated in the future study.

### Disclosure Statement

Dr. D.H. Kim reports no disclosures relevant to the manuscript. Dr. J.S. Jun reports no disclosures relevant to the manuscript. Dr. R. Kim reports no disclosures relevant to the manuscript.

## References

[1] Dunn LT (2002). Raised intracranial pressure. J Neurol Neurosurg Psychiatry.

[2] Juul N, Morris GF, Marshall SB, Marshall LF (2000). Intracranial hypertension and cerebral perfusion pressure: influence on neurological deterioration and outcome in severe head injury. The Executive Committee of the International Selfotel Trial. J Neurosurg.

[3] Balestreri M (2006). Impact of intracranial pressure and cerebral perfusion pressure on severe disability and mortality after head injury. Neurocrit Care.

[4] Raboel PH, Bartek J, Andresen M, Bellander BM, Romner B (2012). Intracranial Pressure Monitoring: Invasive versus Non-Invasive Methods-A Review. Crit Care Res Pract.

[5] Czosnyka M, Pickard JD (2004). Monitoring and interpretation of intracranial pressure. J Neurol Neurosurg Psychiatry.

[6] Kristiansson H (2013). Measuring elevated intracranial pressure through noninvasive methods: a review of the literature. J Neurosurg Anesthesiol.

[7] Newman WD, Hollman AS, Dutton GN, Carachi R (2002). Measurement of optic nerve sheath diameter by ultrasound: a means of detecting acute raised intracranial pressure in hydrocephalus. Br J Ophthalmol.

[8] Bauerle J, Lochner P, Kaps M, Nedelmann M (2012). Intra- and interobsever reliability of sonographic assessment of the optic nerve sheath diameter in healthy adults. J Neuroimaging.

[9] Lochner P (2016). Intra- and interobserver reliability of transorbital sonographic assessment of the optic nerve sheath diameter and optic nerve diameter in healthy adults. J Ultrasound.

[10] Lee JS (2001). Normative measurements of Korean orbital structures revealed by computerized tomography. Acta Ophthalmol Scand.

[11] Maude RR (2013). Transorbital sonographic evaluation of normal optic nerve sheath diameter in healthy volunteers in Bangladesh. PLoS One.

[12] Shirodkar CG (2014). Optic nerve sheath diameter as a marker for evaluation and prognostication of intracranial pressure in Indian patients: An observational study. Indian J Crit Care Med.

[13] Chen H, Ding GS, Zhao YC, Yu RG, Zhou JX (2015). Ultrasound measurement of optic nerve diameter and optic nerve sheath diameter in healthy Chinese adults. BMC Neurol.

[14] Wang L (2016). Ultrasonographic Evaluation of Optic Nerve Sheath Diameter among Healthy Chinese Adults. Ultrasound Med Biol.

[15] Karami M, Shirazinejad S, Shaygannejad V, Shirazinejad Z (2015). Transocular Doppler and optic nerve sheath diameter monitoring to detect intracranial hypertension. Adv Biomed Res.

[16] Rehman H, Khan MS, Nafees M, Rehman AU, Habib A (2016). Optic Nerve Sheath Diameter on Sonography in Idiopathic Intracranial Hypertension Versus Normal. J Coll Physicians Surg Pak.

[17] Ballantyne SA, O’Neill G, Hamilton R, Hollman AS (2002). Observer variation in the sonographic measurement of optic nerve sheath diameter in normal adults. Eur J Ultrasound.

[18] Romagnuolo L, Tayal V, Tomaszewski C, Saunders T, Norton HJ (2005). Optic nerve sheath diameter does not change with patient position. Am J Emerg Med.

[19] Blehar DJ, Gaspari RJ, Montoya A, Calderon R (2008). Correlation of visual axis and coronal axis measurements of the optic nerve sheath diameter. J Ultrasound Med.

[20] Shah S, Kimberly H, Marill K, Noble VE (2009). Ultrasound techniques to measure the optic nerve sheath: is a specialized probe necessary?. Med Sci Monit.

[21] Skoloudik D (2011). Distal enlargement of the optic nerve sheath in the hyperacute stage of intracerebral haemorrhage. Br J Ophthalmol.

[22] Bauerle J (2013). Reproducibility and accuracy of optic nerve sheath diameter assessment using ultrasound compared to magnetic resonance imaging. BMC Neurol.

[23] Strapazzon G (2014). Factors associated with optic nerve sheath diameter during exposure to hypobaric hypoxia. Neurology.

[24] Lochner P (2014). Transorbital sonography in acute optic neuritis: a case-control study. AJNR Am J Neuroradiol.

[25] Amini R, Stolz LA, Patanwala AE, Adhikari S (2015). Coronal Axis Measurement of the Optic Nerve Sheath Diameter Using a Linear Transducer. J Ultrasound Med.

[26] Lefferts WK, Hughes WE, Heffernan KS (2015). Effect of acute high-intensity resistance exercise on optic nerve sheath diameter and ophthalmic artery blood flow pulsatility. J Hum Hypertens.

[27] Goeres P, Zeiler FA, Unger B, Karakitsos D, Gillman LM (2016). Ultrasound assessment of optic nerve sheath diameter in healthy volunteers. J Crit Care.

[28] Zeiler FA (2016). A unique method for estimating the reliability learning curve of optic nerve sheath diameter ultrasound measurement. Crit Ultrasound J.

[29] Dinsmore M, Han JS, Fisher JA, Chan VW, Venkatraghavan L (2017). Effects of acute controlled changes in end-tidal carbon dioxide on the diameter of the optic nerve sheath: a transorbital ultrasonographic study in healthy volunteers. Anaesthesia.

[30] Soldatos T (2008). Optic nerve sonography in the diagnostic evaluation of adult brain injury. Crit Care.

[31] Geeraerts T (2007). Ultrasonography of the optic nerve sheath may be useful for detecting raised intracranial pressure after severe brain injury. Intensive Care Med.

[32] Geeraerts T, Merceron S, Benhamou D, Vigue B, Duranteau J (2008). Non-invasive assessment of intracranial pressure using ocular sonography in neurocritical care patients. Intensive Care Med.

[33] Moretti R, Pizzi B (2009). Optic nerve ultrasound for detection of intracranial hypertension in intracranial hemorrhage patients: confirmation of previous findings in a different patient population. J Neurosurg Anesthesiol.

[34] Moretti R, Pizzi B, Cassini F, Vivaldi N (2009). Reliability of optic nerve ultrasound for the evaluation of patients with spontaneous intracranial hemorrhage. Neurocrit Care.

[35] Lee SU (2016). Optic nerve sheath diameter threshold by ocular ultrasonography for detection of increased intracranial pressure in Korean adult patients with brain lesions. Medicine (Baltimore).

[36] Amini A (2013). Use of the sonographic diameter of optic nerve sheath to estimate intracranial pressure. Am J Emerg Med.

[37] Wang L (2015). Optimal optic nerve sheath diameter threshold for the identification of elevated opening pressure on lumbar puncture in a Chinese population. PLoS One.

[38] Vaiman M, Gottlieb P, Bekerman I (2014). Quantitative relations between the eyeball, the optic nerve, and the optic canal important for intracranial pressure monitoring. Head Face Med.

[39] Vaiman M, Sigal T, Kimiagar I, Bekerman I (2016). Intracranial Pressure Assessment in Traumatic Head Injury with Hemorrhage Via Optic Nerve Sheath Diameter. J Neurotrauma.

[40] Vaiman M, Sigal T, Kimiagar I, Bekerman I (2016). Noninvasive assessment of the intracranial pressure in non-traumatic intracranial hemorrhage. J Clin Neurosci.

[41] De Bernardo M, Rosa N (2017). Clarification on Using Ultrasonography to Detect Intracranial Pressure. JAMA Ophthalmol.

[42] Rosa N, De Bernardo M (2017). Ultrasound assessment of optic nerve sheath diameter in healthy volunteers. J Crit Care.

[43] Iaconetta G, De Bernardo M, Rosa N (2017). Coronal Axis Measurement of the Optic Nerve Sheath Diameter. J Ultrasound Med.

[44] Tenuta M, De Bernardo M, Rosa N (2017). Comments on “Neuromuscular Ultrasonography of Cranial Nerves”. J Clin Neurol.

[45] Rosa N, De Bernardo M (2017). Measurement of the Optic Nerve in a Resource-Limited Setting. J Neurosci Rural Pract.

[46] Caffery TS, Musso MW (2015). Questions regarding the utility of the 30-degree test in measuring optic nerve sheath diameters in ED patients. Am J Emerg Med.

[47] Moosajee M, Restori M, Acheson J, Dahlmann-Noor A (2015). The cost-effectiveness of different strategies to evaluate optic disk drusen in children. J Aapos.

[48] Lagreze WA (2007). Morphometry of the retrobulbar human optic nerve: comparison between conventional sonography and ultrafast magnetic resonance sequences. Invest Ophthalmol Vis Sci.

